# The apolipoprotein B and apolipoprotein A-I Ratio serves as a strong prognostic factor for the overall survival of patients with colorectal cancer

**DOI:** 10.3389/fonc.2022.1089688

**Published:** 2023-01-13

**Authors:** Yangxun Pan, Zhiwei Ye, Yihong Ling, Lingheng Kong, Chenwei Wang, Gong Chen, Desen Wan, Minshan Chen, Dandan Hu

**Affiliations:** ^1^ Department of Liver Surgery, Sun Yat-Sen University Cancer Center, Guangzhou, China; ^2^ Department of Oncology-Pathology, Karolinska Institutet, Solna, Sweden; ^3^ State Key Laboratory of Oncology in South China, Collaborative Innovation Center for Cancer Medicine, Guangzhou, China; ^4^ Department of Pathology, Sun Yat-Sen University Cancer Center, Guangzhou, China; ^5^ Department of Colorectal Surgery, Sun Yat-Sen University Cancer Center, Guangzhou, China

**Keywords:** apolipoprotein B, apolipoprotein A-I, colorectal cancer, prognosis, immunohistochemistry

## Abstract

**Background:**

The lipid metabolism status of patients with colorectal cancer (CRC) has not been understood comprehensively. The present study investigated the characteristics of lipid metabolism parameters in CRC patients with or without metastases and identified the independent prognostic factors of long-term prognosis.

**Methods:**

The clinicopathological data of 231 CRC patients along with 259 formalin-fixed paraffin-embedded samples with or without liver or lung metastasis were retrieved and stained for apolipoprotein B (apoB) *via* immunohistochemistry (IHC) in our center. The correlation and multivariable analysis between blood circulating apolipoprotein A-I (apoA1), apoB and overall survival (OS) were analyzed.

**Results:**

In the multivariable analysis, apoA1, apoB and apolipoprotein B and apolipoprotein A-I (apoB/A) ratio, were identified as independent prognostic factors for OS. Moreover, the apoB/A ratio showed a significantly negative association with OS time (R=-0.187, *P*=0.004). CRC patients with low apoB/A ratio had better 1-, 3- and 5-year OS rates than those who had high apoB/A ratio (87.1%, 54.3%, and 37.1% vs. 92.5%, 72.0%, and 59.5%, respectively, *P*=0.001). On histological level, similar expression intensity of apoB between primary CRC and liver metastases indicated better prognostic outcomes than those with different expression levels (100%, 83.3%, and 77.8% vs. 100%, 66.7%, and 33.3%, respectively; *P*=0.033). Higher level of apoB in the primary CRC interprets into increased incidence of liver metastases. However, the apoB expression levels in the CRC tumor were not parallel to the circulating lipid metabolism parameters.

**Conclusions:**

The apoB/A ratio was a reliable independent prognostic factor for predicting the long-term OS of CRC patients. Moreover, the IHC of the primary CRC and metastatic lesions verified the metastatic potential of apoB through a different aspect. Lipid metabolism status for cancer progression reported in the present study possessed potentially prognostic value, but bench-scale studies are needed for their future clinical applications.

## Background

Colorectal cancer (CRC) ranks as the third most common malignancy in male and the second most common in female (1). In the US, 140,000 new incidences and 50,000 cancer-related deaths of CRC were reported in 2018 (2). After years of development, the overall five-year relative survival can achieve 64.7% in the US, but varies depending on stage of cancer (3). The metabolic syndrome, defined as a cluster of metabolic risk factors that include abdominal obesity, hypertension, hyperglycemia, and dyslipidemia, has been identified as a major risk for CRC according to the epidemiological data worldwide (4, 5). Additionally, in the cases of CRC, liver and lung are the most common distant metastatic sites, which results in the inferior prognosis to others without metastasis (6). However, few studies inspected the relationship between metabolic syndrome, especially lipid metabolism status, and metastatic CRC (mCRC), which might highlight the clues of further understanding CRC and its metastasis. Moreover, identifying the metabolic factors which have significant survival outcomes upon CRC could potentially provide practical dietary instructions to the patients.

In recent years, increasing pre-clinical and clinical studies revealed that metabolism and inflammation played important roles in immune relative therapies (7, 8). Bu et al. introduced the aldolase B as a key regulator in reprogramming CRC to enhance its proliferation in metabolically active organ such as the liver (8). In this perspective, the alternative lipid metabolism into tumor microenvironment has been recently pointed to as a potential fuel of neoplastic cell proliferation, as well as an orchestrator of cancer-related inflammation and immune escape, which caused adverse outcomes (9). Moreover, a recent study highlighted that steatohepatitis could limit the efficiency of immunotherapy for hepatocellular carcinoma patients, which drew lots of interest into the metabolism-related dyslipidemia, especially gastrointestinal tumors that could be affected directly (10). However, the lipid metabolism status among CRC patients with or without metastasis were yet to be fully studied.

In the present study, we retrospectively reviewed the database for patients with CRC or mCRC in our center within the last 10 years. The lipid metabolism status of circulation for the enrolled patients was investigated along with the survival outcomes and available formalin-fixed paraffin-embedded (FFPE) samples. The profiles of immunohistochemistry (IHC) between metastatic lesions and orthotopic colorectal tumors was also checked to comprehensively understand the relationships inside tumors and circulating lipid status. As preliminary results, the ratio of apolipoprotein B and apolipoprotein A-1 (apoB/A) ratio were identified as a reliable prognostic factor for predicting the overall survival (OS) for CRC patients, which provided evidence for individualized treatment strategies in the future.

## Methods

### Patients and clinical information

The CRC database at the Sun Yat-sen University Cancer Center (SYSUCC) was reviewed for CRC patients who received surgical resections with or without metastasis. The clinicopathological characteristics of the patients were obtained from medical records. The follow-up data was performed by consulting the medical records from all hospital departments and the primary care was up to January 1^st^, 2012. The date of cause of death as related to CRC-specific mortality or not were determined from a review of the patient’s files. The venous blood samples were collected before treatment and centrifuged to separate plasma and blood cells. The lipid metabolic parameters, including apolipoprotein A-I (apoA1), high-density lipoprotein-cholesterol (HDL-C), apoB/A ratio, apolipoprotein B (apoB), cholesterol (CHO), low-density lipoprotein-cholesterol (LDL-C) and triglycerides (TG), were collected. Circulation samples were available from 231 patients (87 females and 144 males), and their median age was 57.0 years [interquartile range (IQR), 48.0-64.0 years]. The CRC patients were classified as stage A (n=10), stage B (n=44), stage C (n=53) and stage D (n=124) according to Duke’s staging (11).

This study was approved by the ethical committee of SYSUCC (GZR2019-014) and was conducted consistent with the ethical guidelines of the 1975 Declaration of Helsinki.

### Immunohistochemistry and evaluation

The antibodies that specifically against apoB proteins were performed on FFPE sections for each specimen (Abcam PLC, Discovery Drive, Cambridge Biomedical Campus, Cambridge, CB2 0AX, UK). 4 μm-thick sections cut from the FFPE tissue blocks were deparaffinized and rehydrated using Histo-Clear II (Agar Scientific Ltd, Parsonage Lane, Stansted CM24 8GF, UK) and a graded series of ethanol (Sigma-Aldrich Inc., 3050 Spruce Street, Saint Louis, MO, US; absolute, 99%, 95%, 80%, 70%) for 10 minutes each, followed by 5 minutes washes for twice in phosphate buffered saline (PBS). The following antigen retrieval procedures for antigen were demonstrated in the **Supplementary Materials (** **Table S1** **)**. After retrieval, the sections were washed in PBS for 2×10 minutes and blocked at room temperature for 30 minutes by 3% bovine serum albumin (BSA; Sigma-Aldrich Inc., 3050 Spruce Street, Saint Louis, MO, US). Then the sections were incubated in a humidified chamber overnight at 4°C with the primary antibodies (**Table S1**). Then, the sections were washed in PBS (3×5 minutes) and incubated at room temperature for 30 minutes with the secondary antibody (goat-anti-mouse/rabbit, PV-6000, Zhong-shan Golden Bridge Inc., China). After a wash with PBS (3×5 minutes), colors were developed with the Dako kit (Agilent Technologies, Inc., 5301 Stevens Creek Blvd, Santa Clara, CA 95051, US). The sections were then counterstained with hematoxylin (Sigma-Aldrich Inc., 3050 Spruce Street, Saint Louis, MO, US), dehydrated, and mounted. Negative controls were prepared by substituting PBS for the primary antibodies.

The expression levels were evaluated based on the intensity of the positive signal that was categorized at 3 levels (weak, moderate, and strong). Brown cytoplasmic staining in the colonic carcinoma cells was counted as positive. The results from the IHC-stained slides were independently scored by two histopathologists (YX. P and YH. L) who were blinded to patient information.

### Definitions of outcomes and follow-up after resections

Patients were scheduled to receive dynamic computed tomography (CT) scan or magnetic resonance imaging (MRI), chest radiography and laboratory tests 1 month after treatment, every 3 months during the first 2 years, and every 4-6 months thereafter. If a possibility of progression was suspected based on radiological features such as an abnormality on residual liver or chest, or clinical symptoms (e.g., severe headache or neurological symptoms, bone pain), further tests and treatments would be also performed. OS was defined as the interval between the first treatment and either the date of death or the latest follow-up.

### Statistical analysis

Population demographics, clinical features, and tumor characteristics for included patients were described as median and IQR or percentage according to the nature of the data (**Table 1**). Correlation analysis among interested variables was performed to inspect the associations of variables. Subsequently, univariate and multivariate Cox regression analyses were conducted to determine independently prognostic factors in terms of OS. Hazard ratios (HR) and 95% confidence intervals (95% CI) were also calculated. The entire variables that included in the univariate analyses were inducted into the multivariate analysis using Cox proportional hazards models to investigate the independently prognostic factors. The optimal cutoff value of apoA1, apoB and apoB/A ratio were determined by the maximally selected rank statistics using the “maxstat” package (**Figure S1**). Kaplan-Meier (K-M) curves were applied to estimate all time-to-event functions, and the Log-rank test was calculated for *p*-value. A two-tailed *p*-value<0.050 was considered as statistical significance. All data analyses were performed *via* using R version 4.0.4 statistical software (R Foundation for Statistical Computing, Vienna, Austria).

**Table 1:** Clinical baseline characteristics for enrolled colorectal cancer.

	Overall	apoB/A = Low	apoB/A = High	*P*
N	231	162	69	
Age (median [IQR])	57.00 [48.00, 64.00]	56.00 [47.00, 64.00]	58.00 [49.00, 63.00]	0.823
Gender = Female (%)	87 (37.7)	68 (42.0)	19 (27.5)	0.054
apoB (median [IQR])	0.95 [0.77, 1.12]	0.86 [0.69, 1.00]	1.14 [1.03, 1.33]	<0.001
apoA1 (median [IQR])	1.22 [1.08, 1.39]	1.29 [1.12, 1.46]	1.12 [0.95, 1.22]	<0.001
Surgical resection model (%)				0.041
Orthotopic resection	89 (38.5)	67 (41.4)	22 (31.9)	
Live-metastatic resection	109 (47.2)	68 (42.0)	41 (59.4)	
Lung-metastatic resection	33 (14.3)	27 (16.7)	6 (8.7)	
Cholesterol (median [IQR])	4.97 [4.23, 5.73]	4.79 [4.01, 5.39]	5.54 [4.81, 6.45]	<0.001
HDL-C (median [IQR])	1.20 [0.97, 1.46]	1.30 [1.03, 1.55]	1.04 [0.91, 1.22]	<0.001
LDL-C (median [IQR])	3.07 [2.44, 3.83]	2.85 [2.21, 3.42]	3.84 [3.15, 4.51]	<0.001
Triglycerides (median [IQR])	1.26 [0.92, 1.75]	1.12 [0.88, 1.60]	1.59 [1.10, 1.93]	<0.001
Intensity of apoB (%)				0.408
Non	31 (13.4)	25 (15.4)	6 (8.7)	
Weak	99 (42.9)	70 (43.2)	29 (42.0)	
Moderate	92 (39.8)	62 (38.3)	30 (43.5)	
Strong	9 (3.9)	5 (3.1)	4 (5.8)	
Area of apoB (%)				0.409
Non	31 (13.4)	25 (15.4)	6 (8.7)	
Weak	35 (15.2)	23 (14.2)	12 (17.4)	
Moderate	77 (33.3)	50 (30.9)	27 (39.1)	
Strong	88 (38.1)	64 (39.5)	24 (34.8)	
Differentiations (%)				0.085
Poor	44 (19.0)	25 (15.4)	19 (27.5)	
Moderate	186 (80.5)	136 (84.0)	50 (72.5)	
Well	1 (0.4)	1 (0.6)	0 (0.0)	
Duke’s stage (%)				0.121
A stage	10 (4.3)	10 (6.2)	0 (0.0)	
B stage	44 (19.0)	33 (20.4)	11 (15.9)	
C stage	53 (22.9)	34 (21.0)	19 (27.5)	
D stage	124 (53.7)	85 (52.5)	39 (56.5)	
Death (%)	119 (51.5)	74 (45.7)	45 (65.2)	0.010
Follow-up time (median [IQR])	60.25 [26.75, 67.67]	61.82 [30.11, 68.75]	39.11 [18.21, 65.15]	0.005

Values are presented as the median (interquartile range) or n (%).

apoB/A, apolipoprotein B/apolipoprotein A-I; IQR, interquartile range; OS, overall survival; apoB, apolipoprotein B; apoA1, apolipoprotein A-I; HDL-C, high-density lipoprotein-cholesterol; LDL-C, low-density lipoprotein-cholesterol.

## Results

### Clinical baseline characteristics

**Figure 1:**
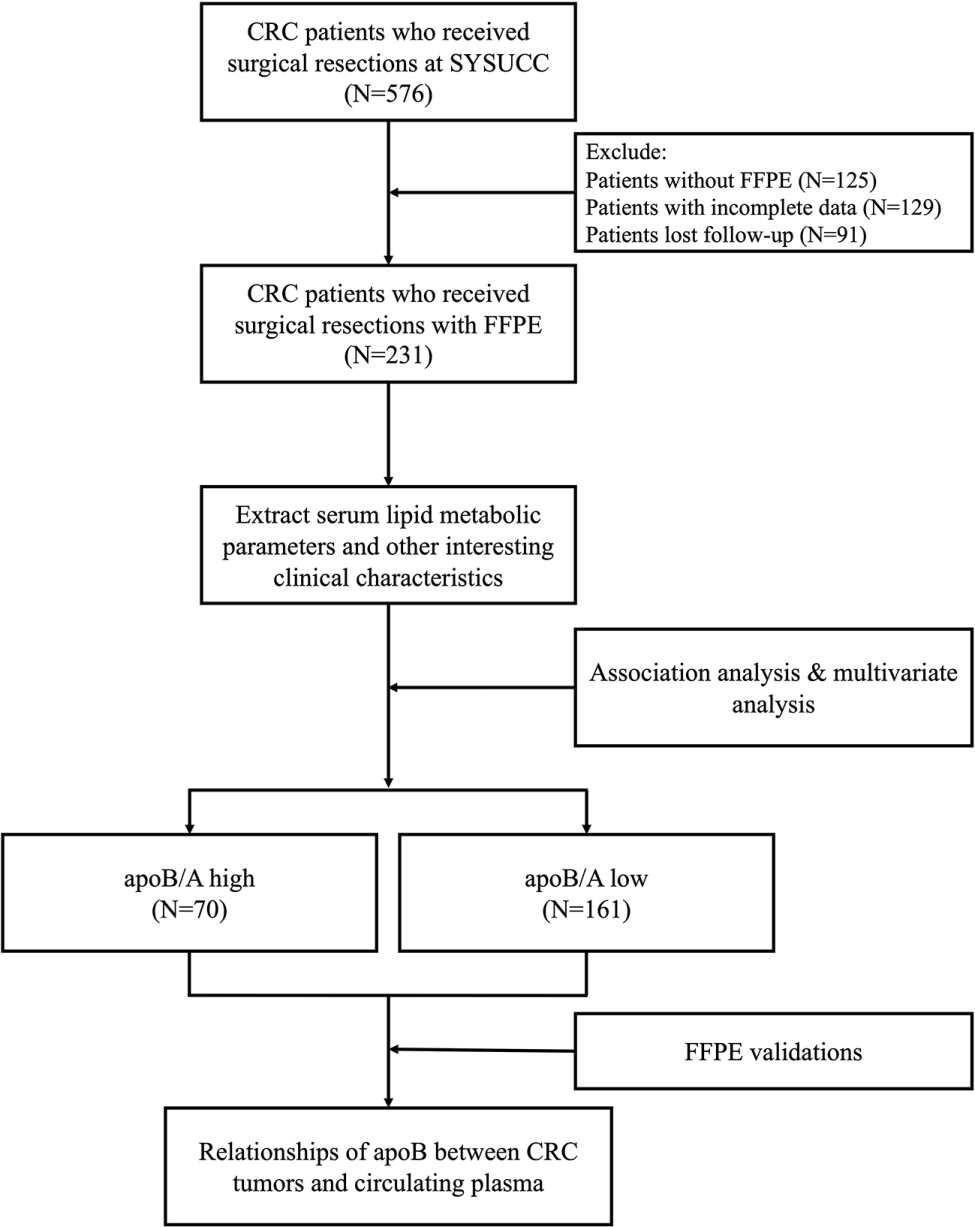
Flow chart for the current study. CRC, colorectal cancer; SYSUCC, Sun Yat-sen University Cancer Center; FFPE, formalin-fixed paraffin-embedded; apoB/A, apolipoprotein B and apolipoprotein A-I ratio; apoB, apolipoprotein B.

Between January 2012 and December 2014, 259 FFPE specimens from 231 CRC patients (27 and 1 patients received colon-liver and colon-lung simultaneous resections, respectively) with complete clinical data and at least 3 years of follow-up for patients who were pathologically diagnosed with colon adenocarcinoma were obtained (**Figure 1**). The clinical baseline characteristics of the total study population were presented in **Table 1**. Out of 231 patients, 119 died because of CRC.

### Associations between lipid metabolism status and clinicopathological variables

**Figure 2:**
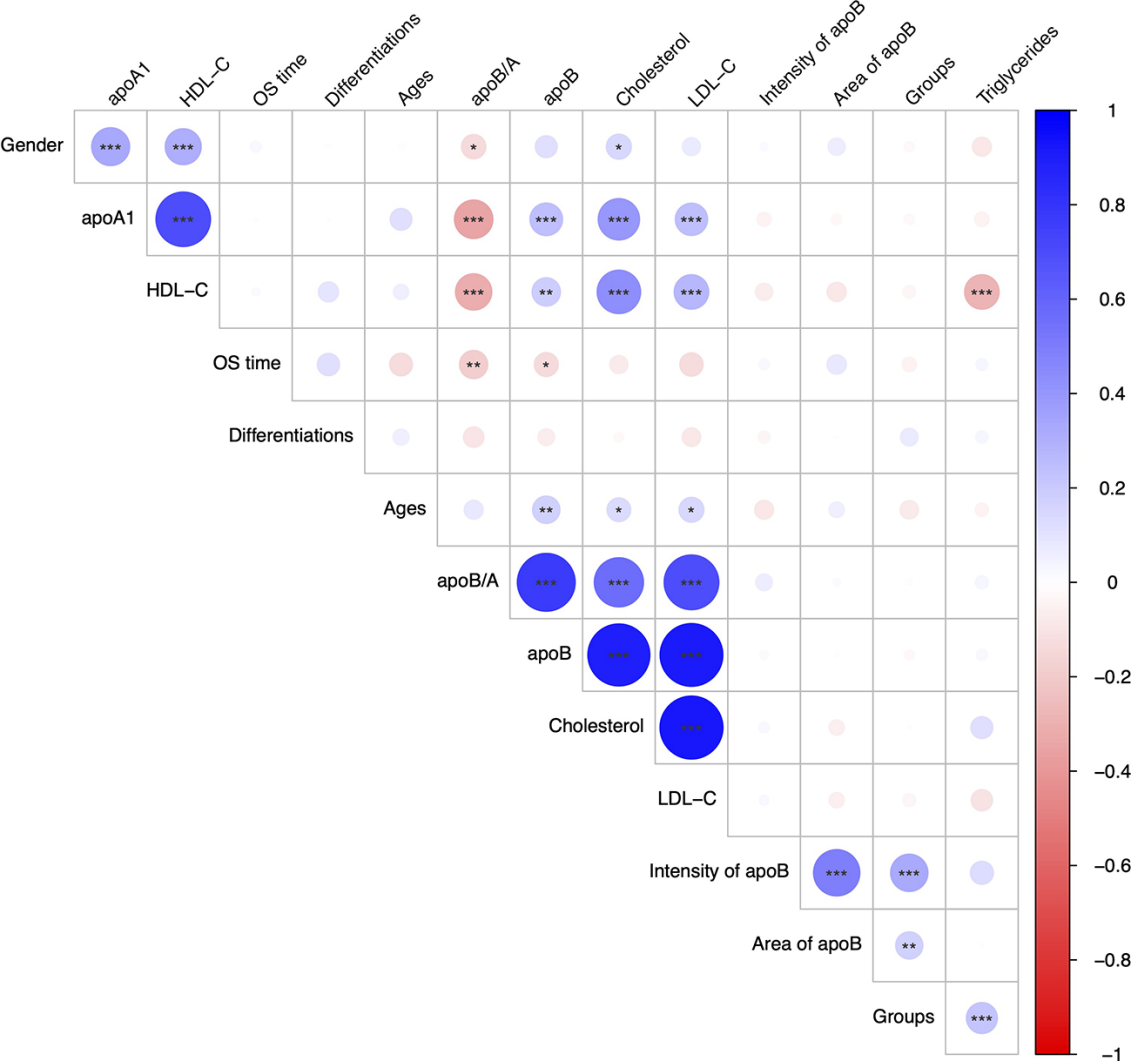
Correlation analysis for the interested clinicopathological parameters.

The relationship between each serum lipid metabolism parameter and clinicopathological variables were demonstrated (**Figure 2**). Generally, majority lipid metabolism parameters, including apoA1, HDL-C, apoB/A ratio, apoB, CHO, LDL-C and TG, presented significantly positive correlations to each other (*P*<0.050). On the contrary, apoB/A ratio demonstrated statistically negative correlation to the HDL-C (*P*<0.050), whereas, it showed statistically positive correlations to CHO and LDL-C (*P*<0.050). Interestingly, apoB/A ratio showed a significantly negative association with OS time (R=-0.187, *P*=0.004), and it indicated that patients with high apoB/A ratios were related to short OS time (**Figure 2**).

### ApoB/A ratio as independent factor for overall survival

**Figure 3:**
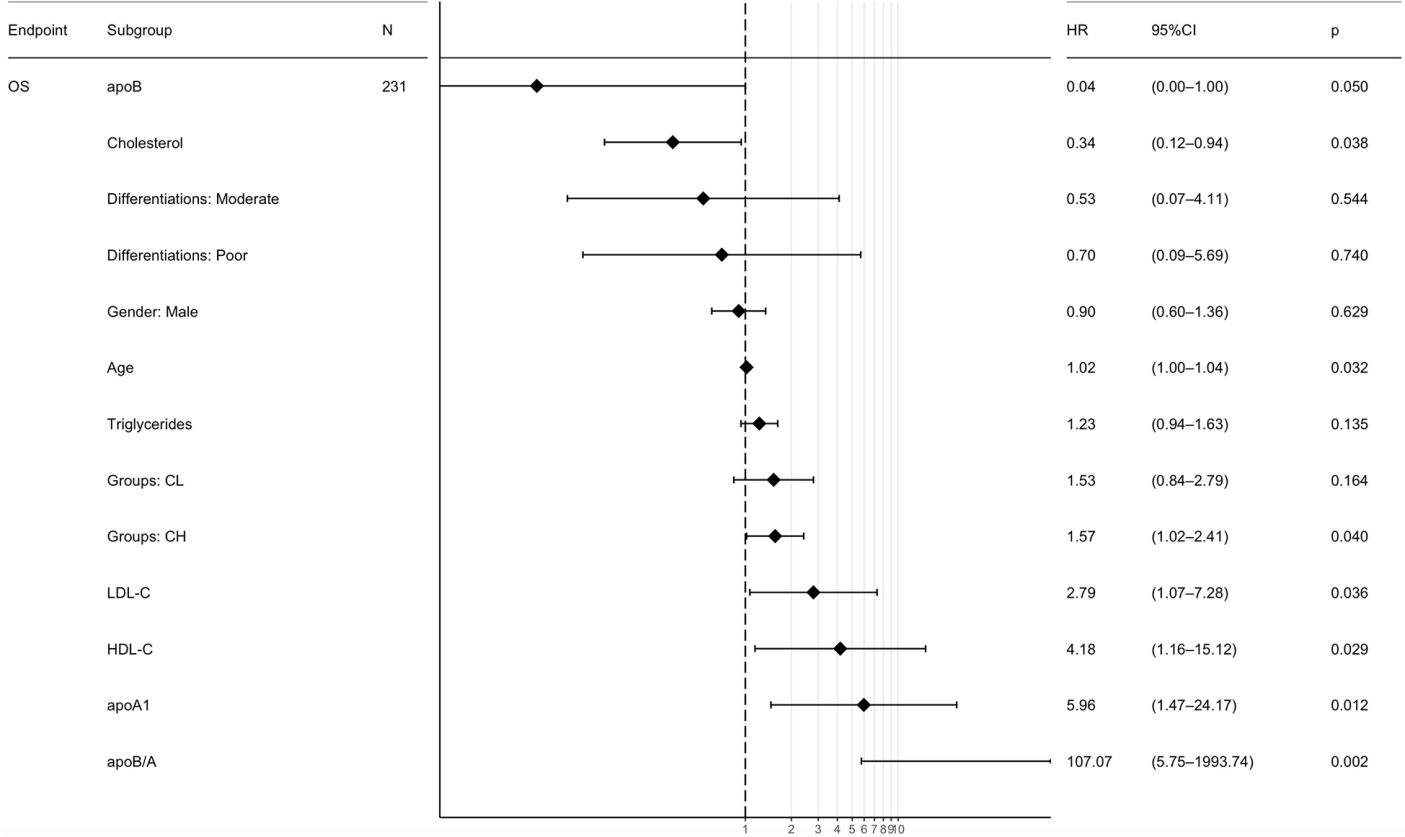
Multivariate Cox regression analyses of the prognostic factors for overall survival. ApoB/A, apolipoprotein B/apolipoprotein A-I; OS, overall survival; CL, colorectal cancer lung metastasis; CH, colorectal cancer hepatic metastasis; apoB, apolipoprotein B; apoA1, apolipoprotein A-I; HDL-C, high-density lipoprotein-cholesterol; LDL-C, low-density lipoprotein-cholesterol.

The lipid metabolism parameters and other interesting variables based on the 231 CRC patients were induced in the Cox univariate analysis before the following multivariate analysis (**Figure S2**). Multivariate analysis revealed that apoB (HR=0.04, 95% CI, 0.00-1.00; *P*=0.050), CHO (HR=0.34; 95% CI, 0.12-0.94; *P*=0.038), Age (HR=1.02; 95% CI, 1.00-1.04; *P*=0.032), CRC with hepatic metastasis (HR=1.57; 95% CI, 1.02-2.41; *P*=0.040), LDL-C (HR=2.79; 95% CI, 1.07-7.28; *P*=0.036), HDL-C (HR=4.18; 95% CI, 1.16-15.12; *P*=0.029), apoA1 (HR=5.96; 95% CI, 1.47-24.17; *P*=0.012) and apoB/A ratio (HR=107.07; 95% CI, 5.75-1993.74; *P*=0.002) were independently prognostic factors for OS (**Figure 3**). Among these lipid metabolism parameters, apoB/A ratio demonstrated the strongest prognostic ability for CRC patients. The univariable analysis were also performed and provided in **Supplementary Materials** (**Figure S2**).

### Overall survival analysis for apoA1, apoB and apoB/A ratio

**Figure 4:**
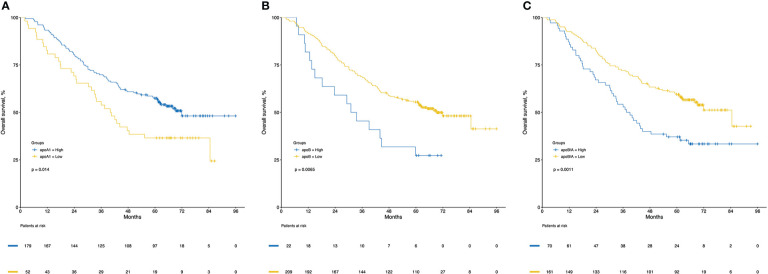
Kaplan-Meier curves of overall survival for apoA1 **(A)**, apoB **(B)** and apoB/A **(C)**. ApoA1, apolipoprotein A-I; apoB, apolipoprotein B; apoB/A, apolipoprotein B/apolipoprotein A-I.

CRC patients were stratified into two groups based on the levels of apoA1, apoB and apoB/A ratio in circulation, respectively, the cutoff values were decided *via* “maxstat” package (**Figure S1**). The low apoA1, high apoB and high apoB/A ratio showed strong discriminative abilities to identify the CRC patients with poor long-term prognosis (*P*<0.050), respectively. Notably, the apoB/A ratio that combined apoA1 and apoB presented the best ability to differentiate the CRC patients into two groups with distinct prognosis, the 1-, 3-, and 5-year OS rates of the high and low apoB/A ratio were 87.1%, 54.3%, and 37.1% vs. 92.5%, 72.0%, and 59.5%, respectively (*P*=0.001, **Figure 4**). Additionally, patients with high apoB/A ratio presented significantly higher percentage of hepatic metastasis in the following subgroup analysis (*P*=0.041; **Table 1**).

**Figure 5:**
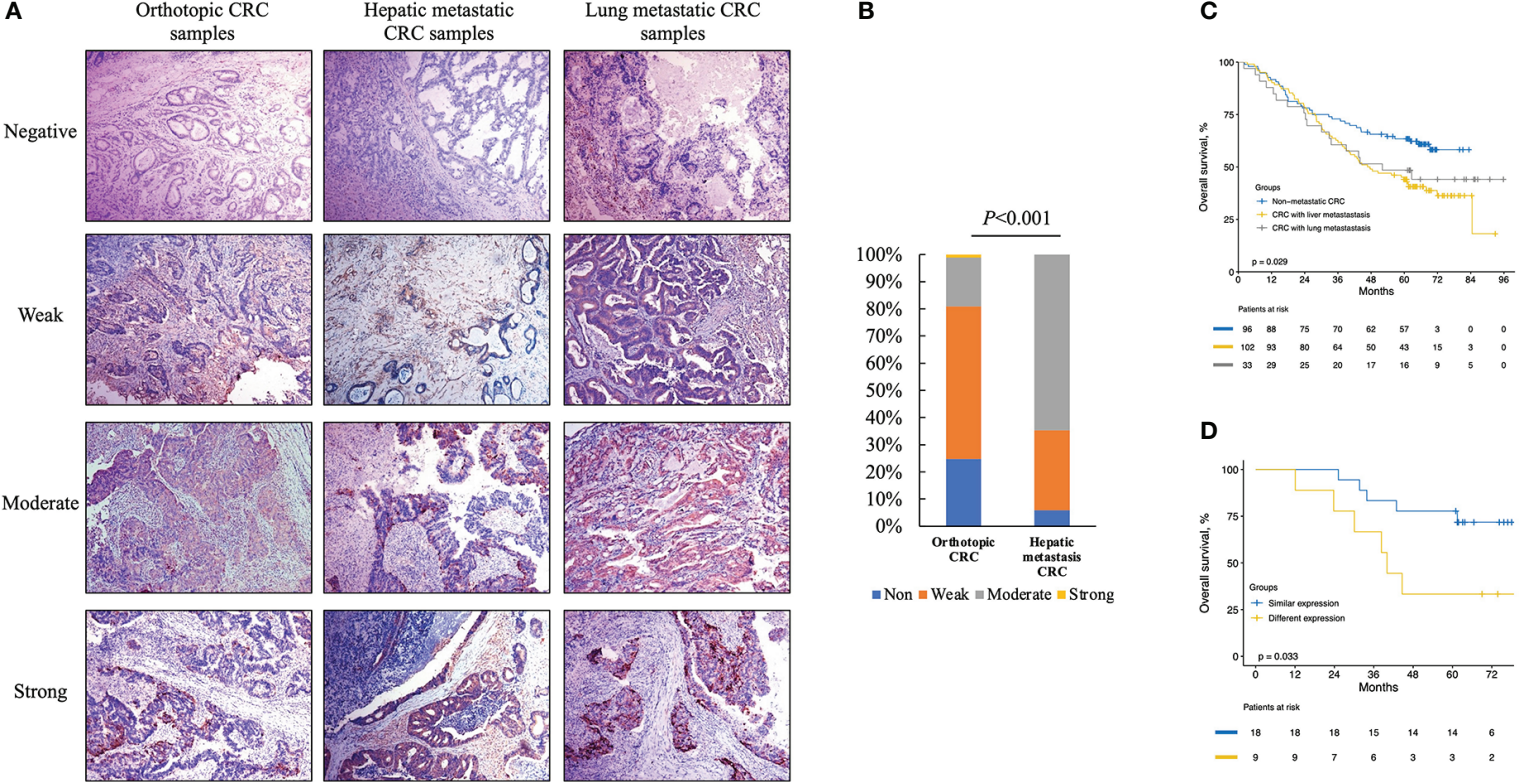
Immunohistochemistry (IHC) for cytoplasm apoB in representative orthotopic and metastatic tissues and the following statistical analysis. **(A)** IHC for cytoplasm apoB in orthotopic CRC, hepatic metastatic CRC and lung metastatic CRC. **(B)** the expression levels of apoB between orthotopic CRC and liver-metastatic specimens. **(C)** the survival analysis according to the non-metastatic CRC, liver-metastatic CRC, and lung-metastatic CRC in 231 patients. **(D)** the survival analysis according to different expression levels between orthotopic and metastatic group in 27 patients received colon-liver simultaneous resection. CRC, colorectal cancer.

Regarding the survival analysis from the entire group, the median follow-up times of 231 patients were 64, 47, and 52 months for non-metastatic CRC patients, liver-metastatic CRC patients, and lung-metastatic CRC patients after surgeries, respectively (**Table 2**). The non-metastatic CRC group had better 1-, 3- and 5-year OS rates than the other two groups (91.7%, 72.9%, and 63.4% vs. 91.2%, 62.7%, and 44.1% vs. 87.9%, 60.6%, and 48.5%, respectively, *P*=0.029; **Figure 5C**).

**Table 2:** The 259 colorectal cancer specimens included in this study.

Characteristics	Overall	Orthotopic samples	Liver-metastatic samples	Lung-metastatic samples	*P*_0_	*P*_1_	*P*_2_	*P*_3_	*P*_4_
Total no. with assessable stains, n	259 (100.0)	124 (47.9)	102 (39.4)	33 (12.7)					
Sample Source, n (%)					<0.001				
Colon cancer	124 (47.9)	124 (100.0)	0 (0.0)	0 (0.0)					
Liver metastasis	102 (39.4)	0 (0.0)	102 (100.0)	0 (0.0)					
Lung metastasis	33 (12.7)	0 (0.0)	0 (0.0)	33 (100.0)					
Apolipoprotein B, n (%)					0.012	0.002	0.475	0.996	0.014
Negative	33 (12.7)	24 (19.4)	6 (5.9)	3 (9.1)					
Weak	107 (41.3)	61 (49.2)	33 (32.4)	13 (39.4)					
Moderate	110 (42.5)	38 (30.6)	57 (55.9)	15 (45.5)					
Strong	9 (3.5)	1 (0.8)	6 (5.9)	2 (6.1)					
Differentiation, n (%)					0.7797	0.9912	0.5921	-	0.759
Poor	51 (19.7)	27 (21.8)	22 (21.6)	2 (6.1)					
Moderate	207 (79.9)	96 (77.4)	80 (78.4)	31 (93.9)					
Well	1 (0.4)	1 (0.8)	0 (0.0)	0 (0.0)					

P_0_, comparison among three groups; P_1_, comparison between colon cancer and liver metastasis; P_2_, comparison between colon cancer and lung metastasis; P_3_, comparison between liver.

metastasis and lung metastasis; P_4_, comparison between colonic cancer and both metastases. CRC, colorectal cancer.

### Relationships of apoB between CRC tumors and circulation

Based on the discriminative ability of circulating apoB in stratifying the CRC patients into significantly different prognosis, the apoB expression level in CRC and its metastasis were further investigated. The apoB was selected to primarily detected the protein levels in the cytoplasm of CRC cells according to previous study and the representative pictures were showed (**Figure 5A**) (12). Surprisingly, apoB showed a significant liver-metastatic specificity (55.9% vs. 30.6% of moderate expression, *P*=0.002) compared to the corresponding orthotopic CRC (**Table 2**). Regarding to the orthotopic CRC, the expression levels of apoB were higher in the liver-metastatic CRC than the CRC patients who did not present metastasis (22 and 0 out of 34 vs. 16 and 1 out of 89 for medium and strong expression, respectively, *P*<0.001, **Figure 5B**). Furthermore, in general, higher apoB levels were observed in the liver and lung metastasis compared to that observed in the orthotopic CRC (72 and 8 of 135 vs. 38 and 1 of 124, respectively, *P*=0.014; **Table 2**). Moreover, the intensity and area of apoB had significant associations with the hepatic metastasis, respectively (R=0.327, *P*<0.001; R=0.174, *P*=0.008, respectively; **Figure 2**). Interestingly, for the 27 liver-metastatic CRC patients who had availably both orthotopic and matched liver-metastatic CRC specimens, the specimens were divided into two groups according to the difference of apoB expression levels between orthotopic CRC and matched hepatic metastasis. The identical apoB expression levels between orthotopic CRC and the metastatic group had significantly higher 1-, 3- and 5-year OS rates than those presented differently expression-level group (100%, 83.3%, and 77.8% vs. 100%, 66.7%, and 33.3%, respectively; **Figure 5D**, *P*=0.033). However, a larger volume of samples was needed to validate these findings in the future.

## Discussion

In the present study, we screened circulatory samples from 231 CRC patients with or without metastasis for the lipid metabolism parameters along with the IHC staining for apoB in the tumor tissues. This study inspected the lipid metabolism status in circulation and tumor tissues simultaneously, and apoB/A was determined as a reliable marker that were associated with CRC long-term prognosis.

Unlike many other cancers, the vast majority of CRC were thought to evolve from conventional adenomas which underwent dozens of mutations, and this process was referred to as the adenoma-to-carcinoma procession (13). And it was commonly recognized that the development of CRC accumulated a series of mutations to gain metastatic competence, including the ability to proliferate, invade into the surrounding tissues, survive in the circulatory system, colonize distant organs and eventually resume growth (14). Several oncogenes including the Kras with Apc and Tp53 deficiency were sufficient to enhance proliferation in animal experiment (15, 16). It was commonly accepted that cancer cells possessed the unique ability to generate energy in a nutrient-deficient tumor microenvironment, and they demonstrated a preference of glycolysis rather than oxidative phosphorylation (OXPHO) when oxygen was sufficient, which had been verified by Otto Warburg (17). Therefore, the intensively proliferating cancer cells could display unique metabolic patterns based on which they might obtain enough energy for new biomass synthesis (17).

The lipidomics which was a distinct branch of metabolome studies provided information with respect to the role of lipid dysregulation in various pathological conditions, and it had been proven to be a critical role in various malignancies (18–20). The lipid composition and metabolism were highlighted in carcinoma metabolism in recent years, especially in CRC patients (21). ApoA1 and apoB were the two major sources of apolipoproteins involved in lipid transport and were proven to function in the procession causing atherosclerosis and its complications (22). ApoA1 was reported to be the major protein in high-density lipoprotein (HDL) particles, while the apoB was the major protein in very low-density lipoproteins (VLDL), intermediate-density lipoproteins (IDL) and low-density lipoproteins (LDL) (23). Whereas the research that investigating the lipid metabolism status of the circulating for CRC patients were still absence.

In the present study, although the circulatory apoA1 and apoB were identified as independent prognostic factors of OS for CRC patients, the apoB/A ratio further enhanced the predictive ability for long-term survival of these patients. In addition, the following analysis partially explained the reasons why CRC patients with high apoB/A suffered from poor long-term prognosis. There were higher percentages of hepatic metastasis in the high apoB/A ratio group, which was thought to be the prognostic factor that mainly decrease the OS in other study (24). The comparison between the two groups also revealed that the high apoB/A ratio group was at the higher levels of lipid metabolism status than those of the low apoB/A ratio group, including higher LDL-C, lower HDL-C, higher CHO and higher TG which were thought to be clinical manifestations of metabolic syndrome (5) (**Table 1**). A previous study reported that the concentrations of circulatory lipoproteins and free fatty acids could be affected by the intake of fatty acids, and this affect might be one possible reason to explain the principle lipid metabolic activity for CRC patients with high apoB/A ratio, since the dietary habit was one of the critical risk factors for CRC (25, 26). However, this hypothesis needs to be further validated in the future.

With respect to the apoB expression levels in CRC, neither the intensity nor area of apoB demonstrated associations to the circulating lipid metabolism parameters (**Figure 2**). This result indicated that CRC contributed little to the lipid metabolic activity in circulation. Noticeably, apoB, on the other hand, was identified as playing a crucial role in the specificity of organ distribution, and we observed significant differences in apoB expression levels between the orthotopic CRC and liver-metastatic CRC. Since apoB gene encodes a major component of lipoprotein that was important in lipid transfer and lipoprotein assembly, previous study also reported apoB showed correlations with liver-specific metastasis (27, 28). As the role of metabolic reprogramming had been described in CRC liver metastasis in a previous study, it was also noted that the metabolic change could be regulated by apoB expression levels between CRC and mCRC, which might result in worse long-term outcomes (8). Moreover, the metabolic reprogramming *via* mutation or downregulation of hypermethylated genes, including apoB and albumin, was observed in hepatocellular carcinoma, which indicated that this genetic pattern of metabolism-related regulation was possible to promote the progression in malignant tumors (29). These results additionally provided clues for further studies in terms of CRC lipid metastatic metabolism.

**Figure 6:**
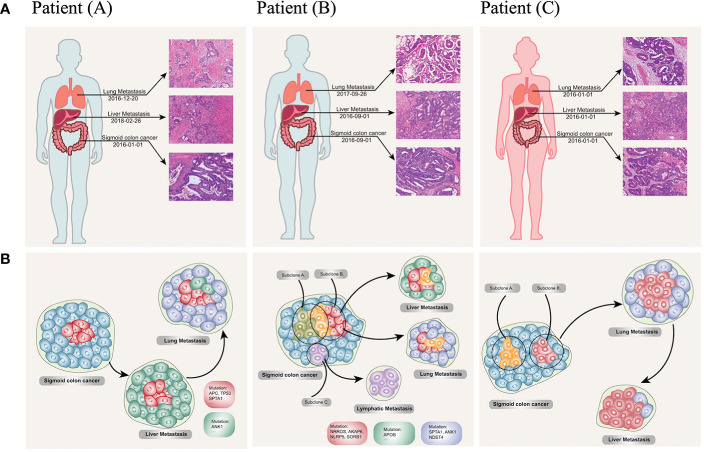
Clinical, pathological **(A)** and phylogenetic tree **(B)** of the three multiorgan metastatic patients. **(A)** Representative H&E-stained histological sections of primary colon cancer, lymph node, liver metastases, and lung metastases. (10 × 20 magnification). Patient **(A)** developed synchronous liver metastases at the diagnosis of the sigmoid colon cancer and revealed lung metastases one year after the resection of colonic and hepatic lesions; patient **(B)** reported lung lesion one year after the initial Dixon surgery, and intrahepatic metastases after the pulmonary lobectomy; patient **(C)** presented synchronous liver and lung metastases at her initial diagnosis. **(B)** The original subclone (red cells and cyan cells) contains driver mutations (TP53, APC, KRAS), which remain unchanged during the disease course. Novel mutations in the hepatic (green cells) and pneumonic (blue cells) metastases could pass on to the other site of metastases according to their expansion process. Lymphatic metastases seed from a distinct subpopulation of primary tumor (purple cells). Reconstructed cancer phylogenies demonstrate patient **(A)**. Original colon cancer seeds liver metastases, which in turn leads to subsequent lung lesion; patient **(B)**. a small genetic variant from subclone A to subclone B give rise to independent metastases to the liver and lung, while a distinct subclone in the primary tumor moves to the reginal lymph node; patient **(C)** heterogeneity exists in the primary colon tumor, one of the subclone seeds the lung nodule before it evolves to the liver.

Comprehensively understanding the rules of the multiorgan metastasis model for CRC still was a long way to undertake. We also preliminarily reviewed three multiorgan metastasis CRC patients *via* the whole exome sequencing (WES) based on our unpublished data. The results indicated a variety of relationships among the orthotopic and metastatic CRC (**Figures 6A**, **4B**). Our initial outcomes were able to provide inspiring insights into the development, progression and metastasis of cancer, especially with respect to organ-dependent metastasis. Encouragingly, the apoB was also found as a specifically significant mutation in the patient B who developed with hepatic metastasis (**Figure 6B**). Furthermore, from a perspective of personalized diagnosis and therapies, apoB and apoA were able to serve valuable information in terms of decision-making. CRC patients with higher apoB/apoA ratio tended to suffer from worse prognosis and needed to perform intensive therapies including adjuvant treatment and high-end imaging follow-up. Additionally, when taking the IHC results into consideration, high expression of apoB in the CRC tumor tissue might indicate the possibility of liver metastasis, and this request for liver-specific surveillance during the following follow-up for patients who had this characteristic. Since the genetic testing became an increasing important assistance for treatment determination for patients with CRC, comprehensive evaluations taking both genetics and protein aspects would be highly recommended for the multidisciplinary treatment. The KRAS mutations in mCRC had proven its prognostic values and associations with the cholesterol to high-density lipoprotein ratio, and it indicated that mCRC patients treated with lipid lowering therapies might benefit more if they presented KRAS mutated (30). Therefore, the prospective investigation from genetic and lipoprotein levels was able to understand the CRC biological behaviors comprehensively and develop the individualized treatment plan. The present study provided preliminary clues to the non-random dissemination of metastases, and the causality of this correlation still needed verification in the future.

There were several limitations identified in the present study that needed to be taken into consideration. First, the included population was relatively small influencing the statistical evaluation and especially associations with stage, which was weak due to low power. This also made it hard to stratify the patients with respect to more variables such as etiology, type of surgery, obesity. Second, there was a selective bias for the studied subjected because of the retrospective and single-center nature of this study. Third, it was also important to point out that the levels of lipid metabolism in the circulation might reflect the release of factors due to other underlying diseases or by systemic inflammations in general other than cancer activities. And in the present study, we did not have the possibility to investigate this.

## Conclusions

The current study identified that apoB/A ratio could reflect the lipid metabolism status and was a reliable independently prognostic factor for predicting long-term OS of CRC patients. Moreover, the IHC verification for apoB in the CRC tissue provided a preliminary result for the relationship between tumor and circulation. Lipid metabolism status for cancer progression reported in the present study possessed potentially prognostic value, but bench-scale studies were needed for their future clinical application.

## Data availability statement

The original contributions presented in the study are included in the article/**Supplementary Materials**, further inquiries can be directed to the corresponding author/s.

## Ethics statement

The studies involving human participants were reviewed and approved by ethical committee of SYSUCC (GZR2019-014). The patients/participants provided their written informed consent to participate in this study.

## Author contributions

YP, ZY, YL and DH designed experiments and drafted the manuscript. CW, LK, GC revise the manuscript. MC, DW and DH approved the final version. All authors contributed to the article and approved the submitted version.

## Supplementary material

### Abbreviations

CRC, colorectal cancer; apoB, apolipoprotein B; apoA1, apolipoprotein A-I; apoB/A, apolipoprotein B and apolipoprotein A-I; OS, overall survival; FFPE, formalin-fixed paraffin-embedded; IHC, immunohistochemistry; SYSUCC, Sun Yat-sen University Cancer Center; HDL-C, high-density lipoprotein-cholesterol; LDL-C, low-density lipoprotein-cholesterol; CHO, cholesterol; TG, triglycerides; PBS, phosphate buffered saline; CT, computed tomography; MRI, magnetic resonance imaging; IQR, interquartile range; HR, hazard ratios; CI, confidence intervals; OXPHOs, oxidative phosphorylations.
