# Supplementary material for: The apolipoprotein B and apolipoprotein A-I Ratio serves as a strong prognostic factor for the overall survival of patients with colorectal cancer
Source: Front Oncol. 2023 Jan 13;12:1089688. doi: 10.3389/fonc.2022.1089688 (PMC9880464; doi:10.3389/fonc.2022.1089688)
Supplement: Supplementary file 1 [file DataSheet_1.pdf]

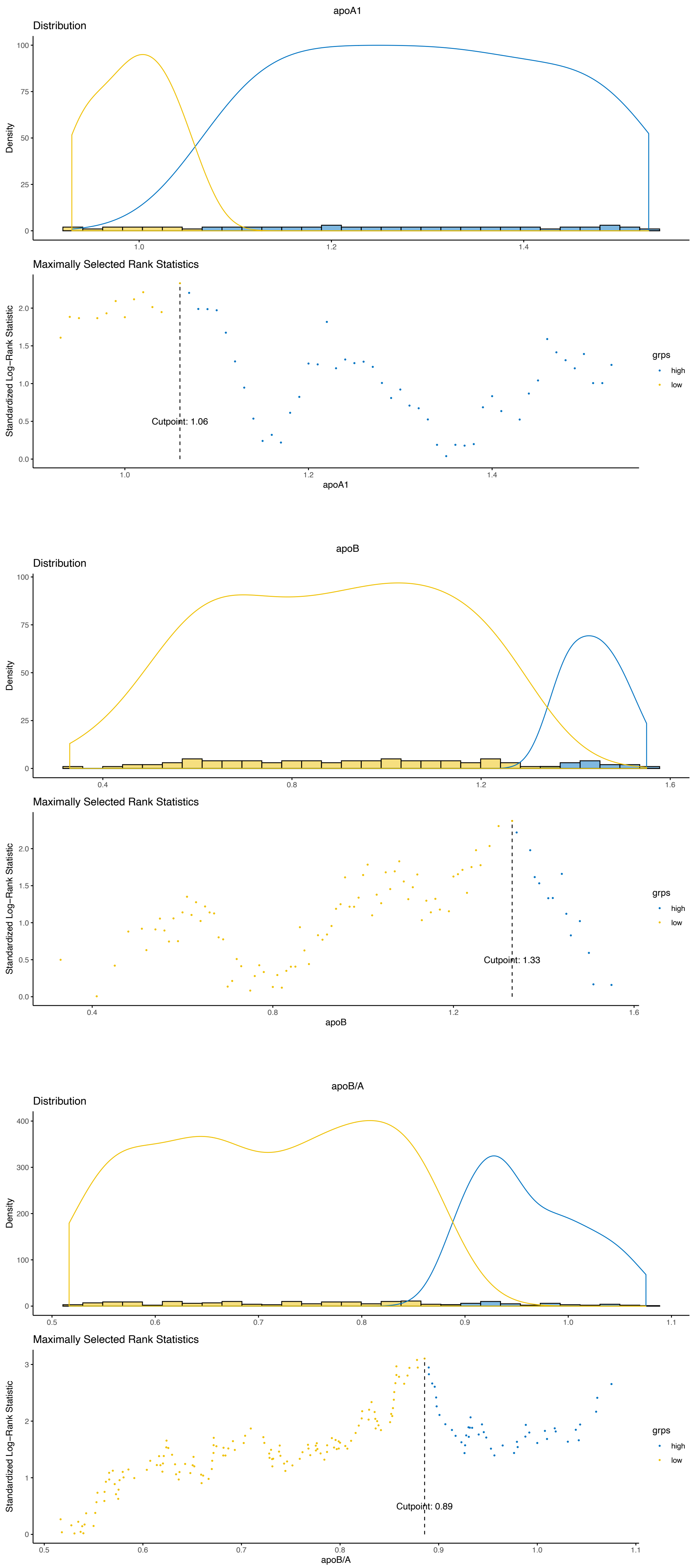

Figure S1 Cutoff values of apoA1, apoB and apoB/A depended on the ‘maxstat’ package. ApoB/A, apolipoprotein B/apolipoprotein A-I; apoB, apolipoprotein B; apoA1, apolipoprotein A-I.

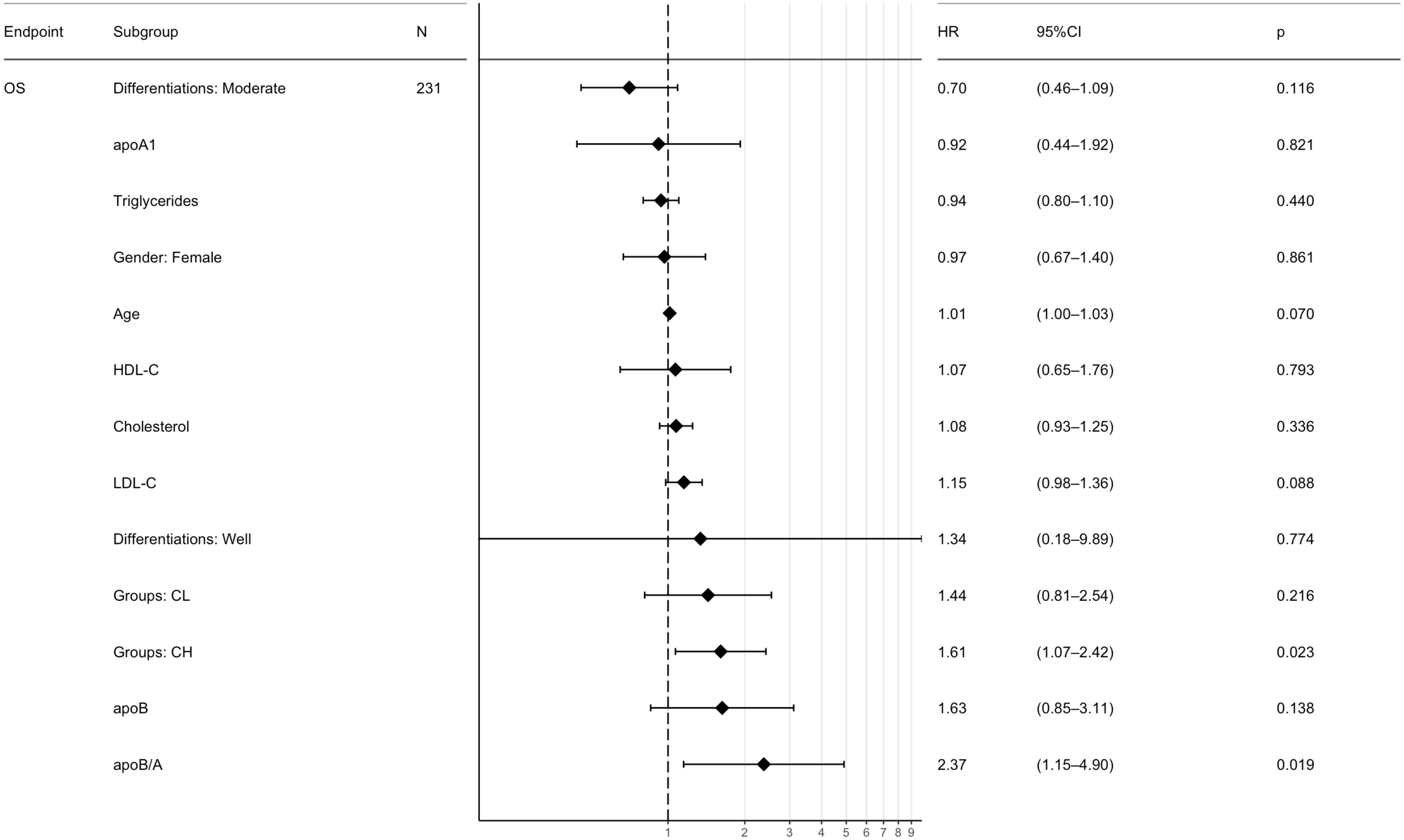

Figure S2 Univariate cox regression analyses of the prognostic factors for overall survival. ApoB/A, apolipoprotein B/apolipoprotein A-I; OS, overall survival; CL, colorectal cancer lung metastasis; CH, colorectal cancer hepatic metastasis; apoB, apolipoprotein B; apoA1, apolipoprotein A-I; HDL-C, high-density lipoprotein-cholesterol; LDL-C, low-density lipoprotein-cholesterol.

Table S1. List of primary antibodies used for immunohistochemical staining.

Antibody name	Manufacturer	Host	Retrieval methods	Dilution	Development (minute)
Anti-Apolipoprotein B antibody	Abcam PLC, ab20737	Rabbit	EDTA, PH=8.0	1:400	2:30
