# Supplementary figures and images for: The apolipoprotein B and apolipoprotein A-I Ratio serves as a strong prognostic factor for the overall survival of patients with colorectal cancer
Source: Front Oncol. 2023 Jan 13;12:1089688. doi: 10.3389/fonc.2022.1089688 (PMC9880464; doi:10.3389/fonc.2022.1089688)

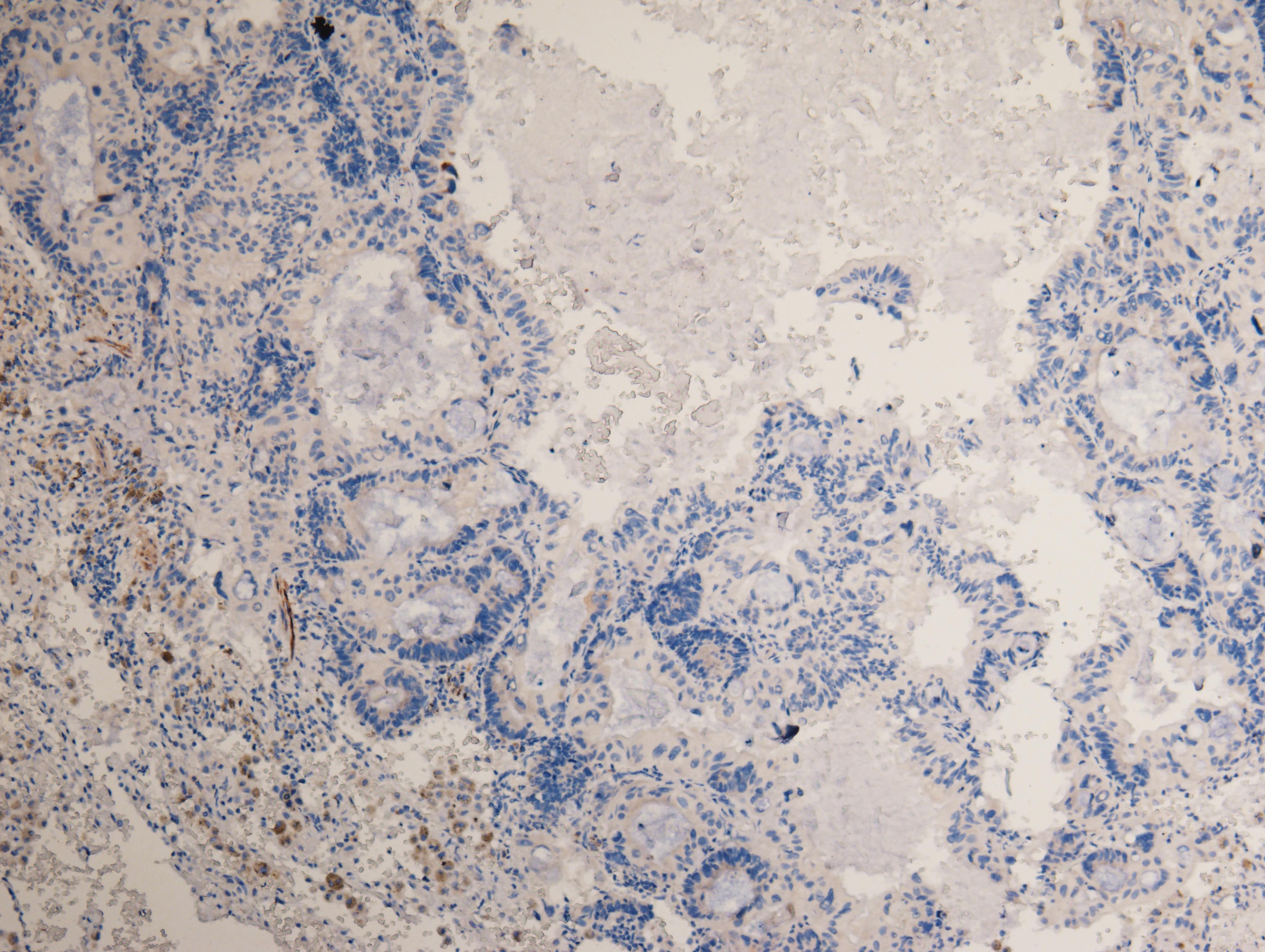

Supplement: Supplementary file 3 [file DataSheet_3.zip › OriginalImages/-F-100x-1.jpg]

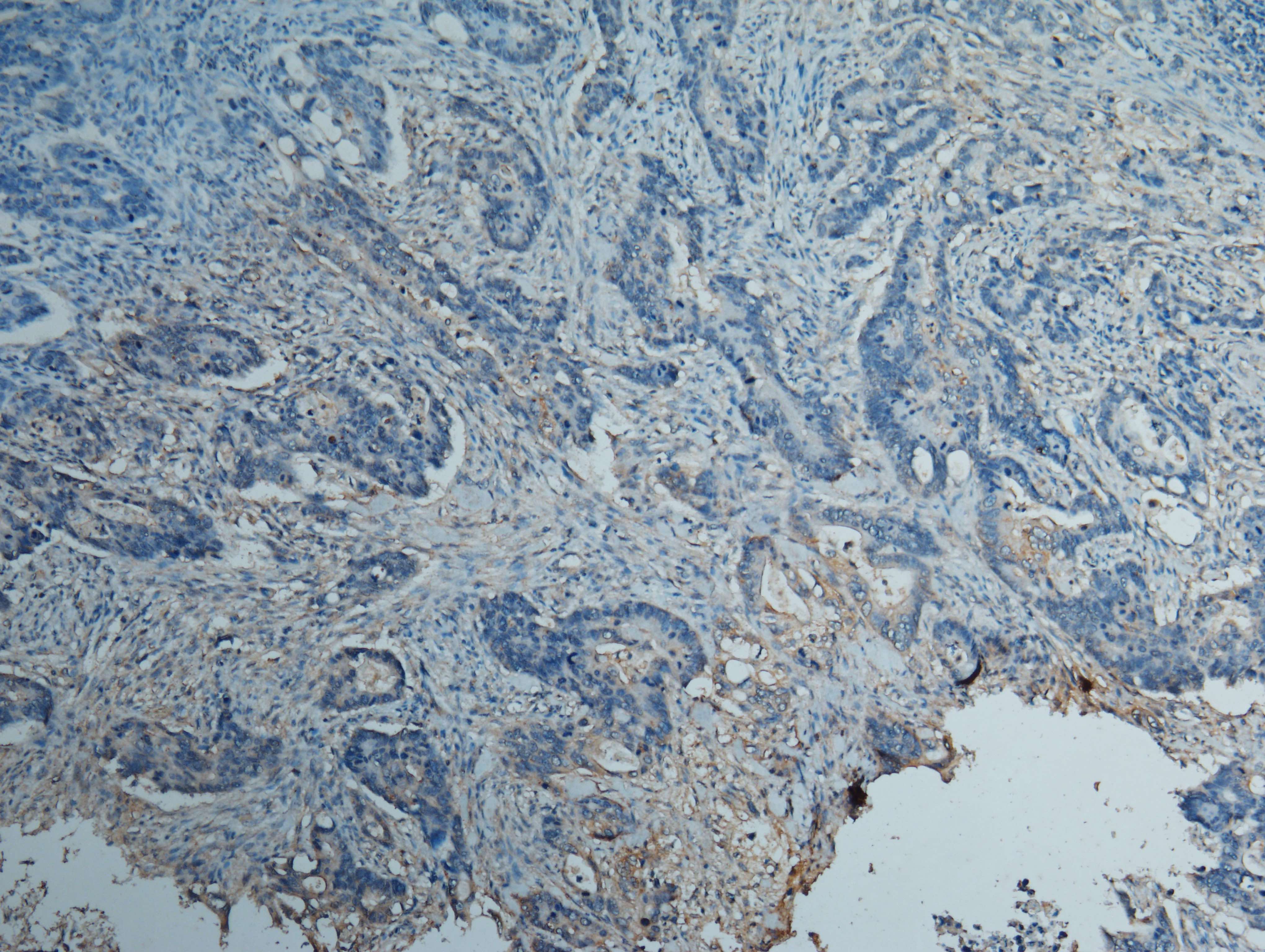

Supplement: Supplementary file 3 [file DataSheet_3.zip › OriginalImages/1-C-100x-2.jpeg]

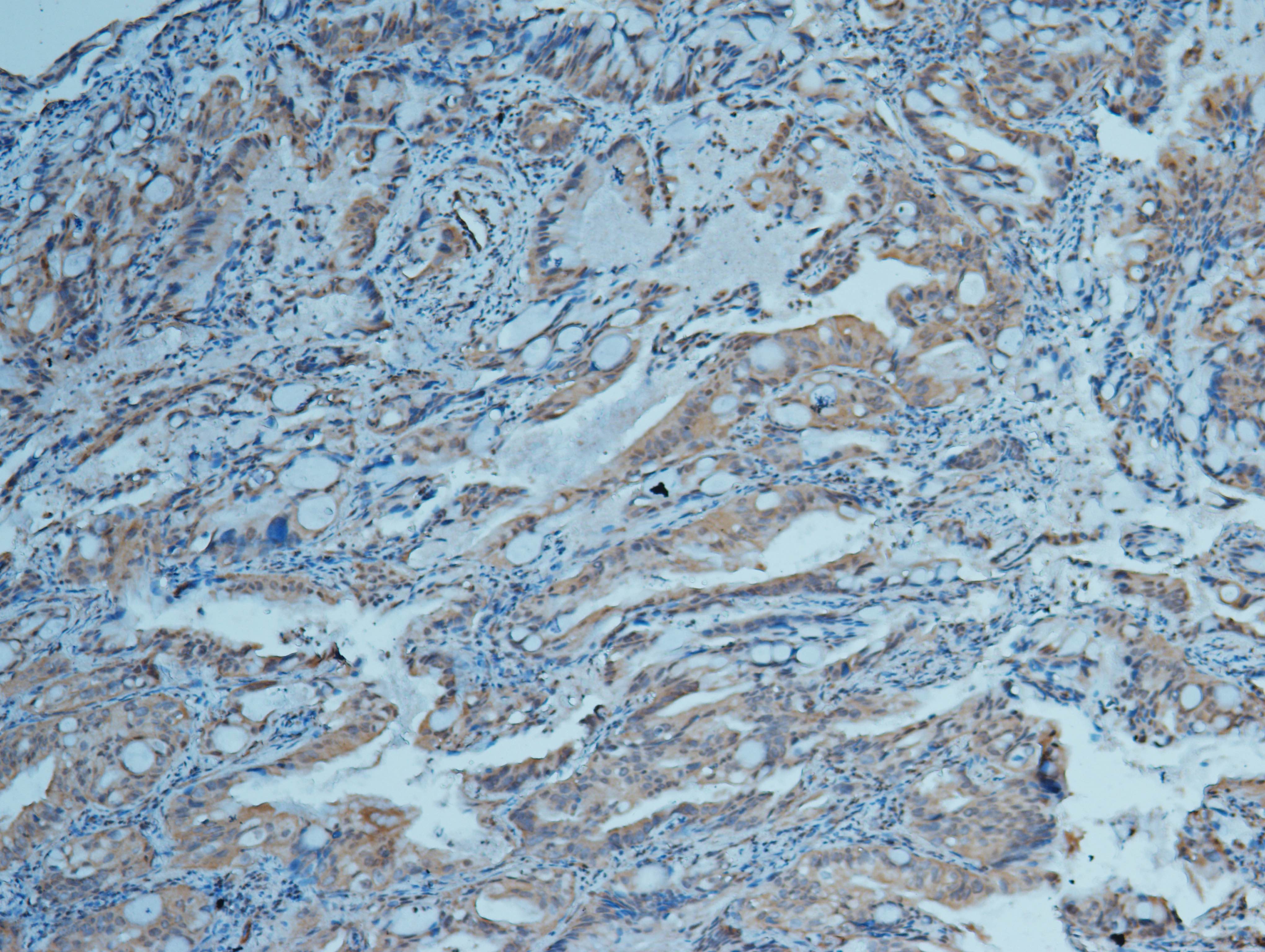

Supplement: Supplementary file 3 [file DataSheet_3.zip › OriginalImages/2-F-100x-2.jpeg]

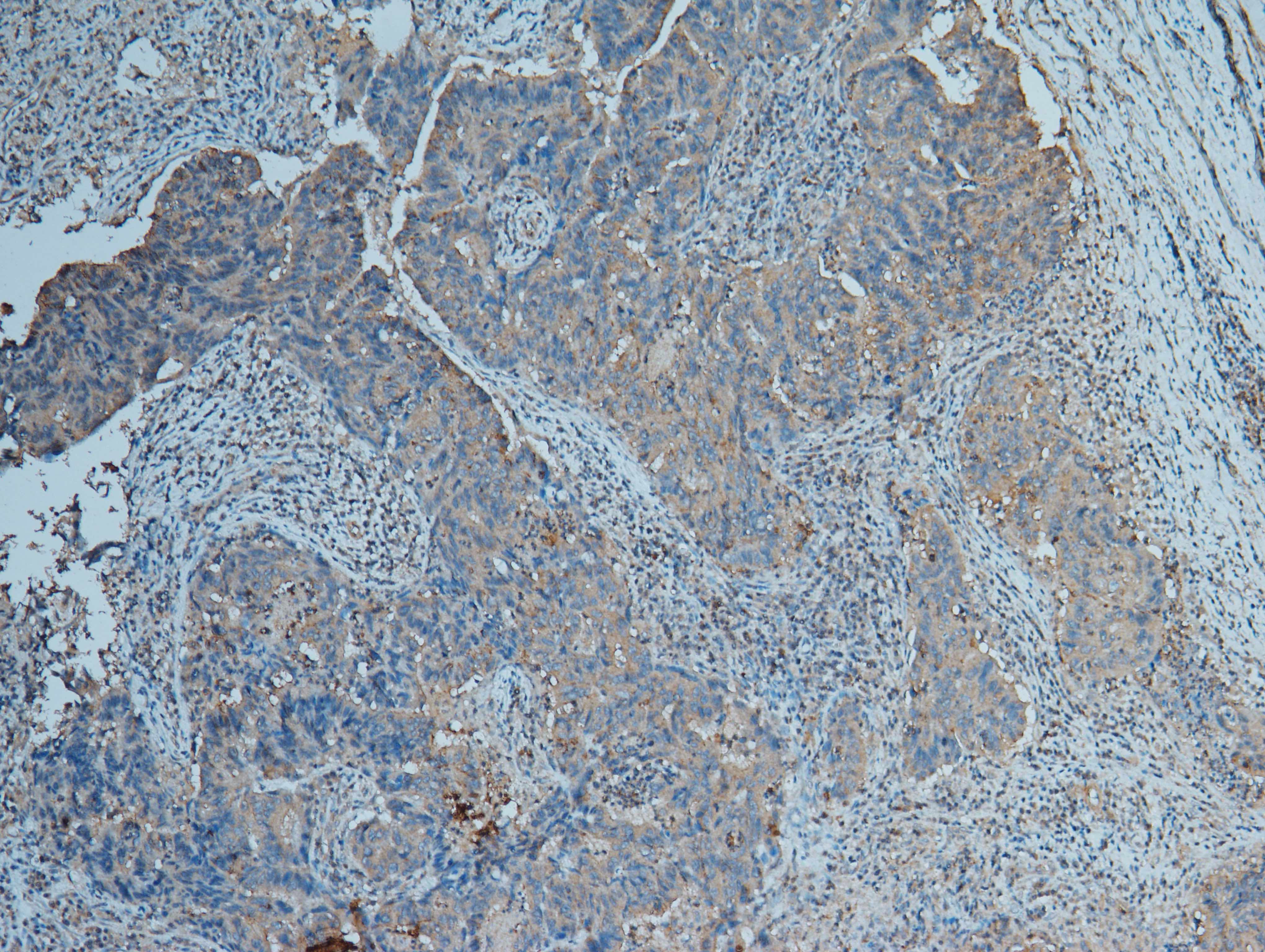

Supplement: Supplementary file 3 [file DataSheet_3.zip › OriginalImages/2-C-100x-2.jpeg]

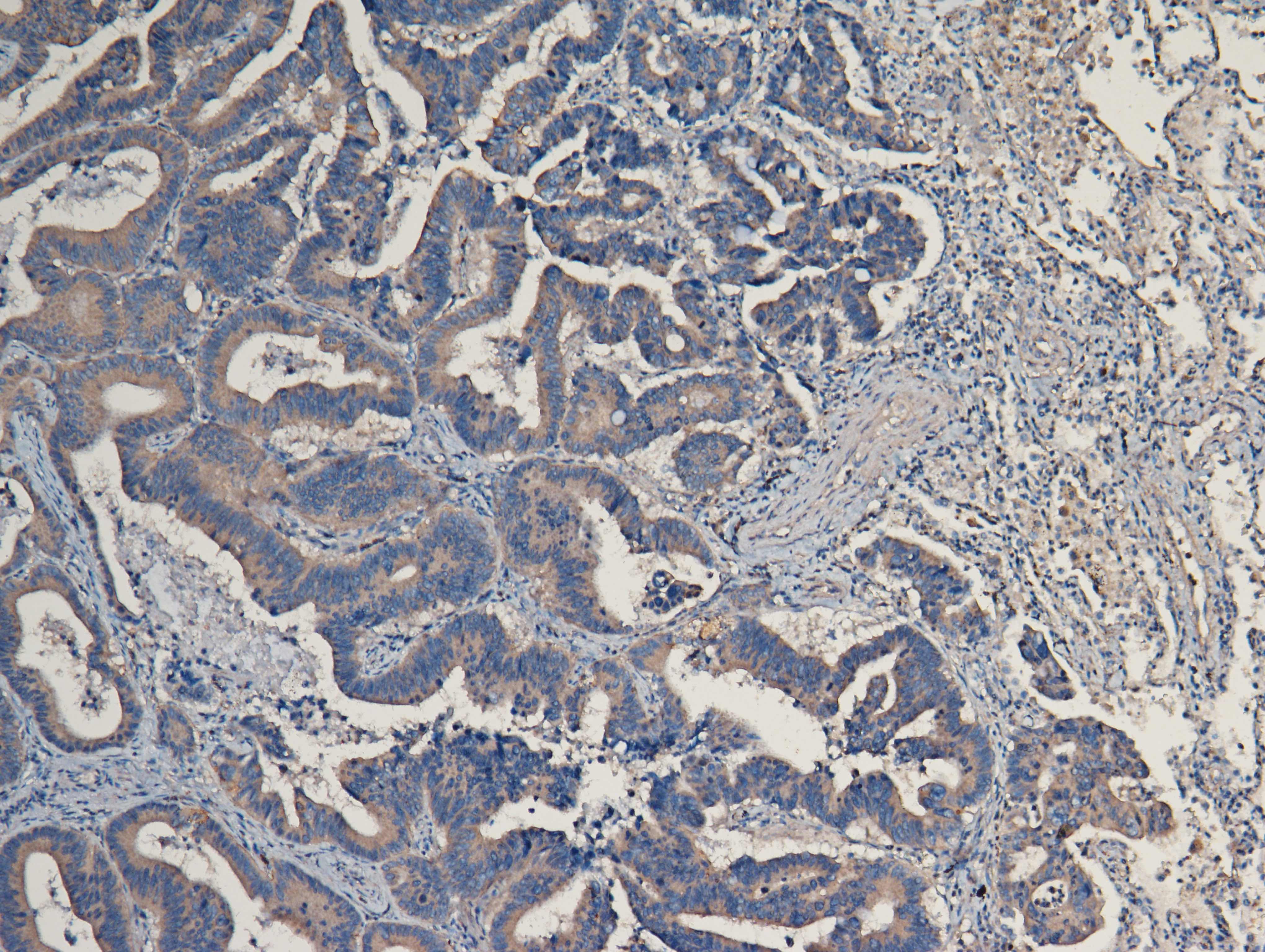

Supplement: Supplementary file 3 [file DataSheet_3.zip › OriginalImages/1-F-100x-2.jpeg]

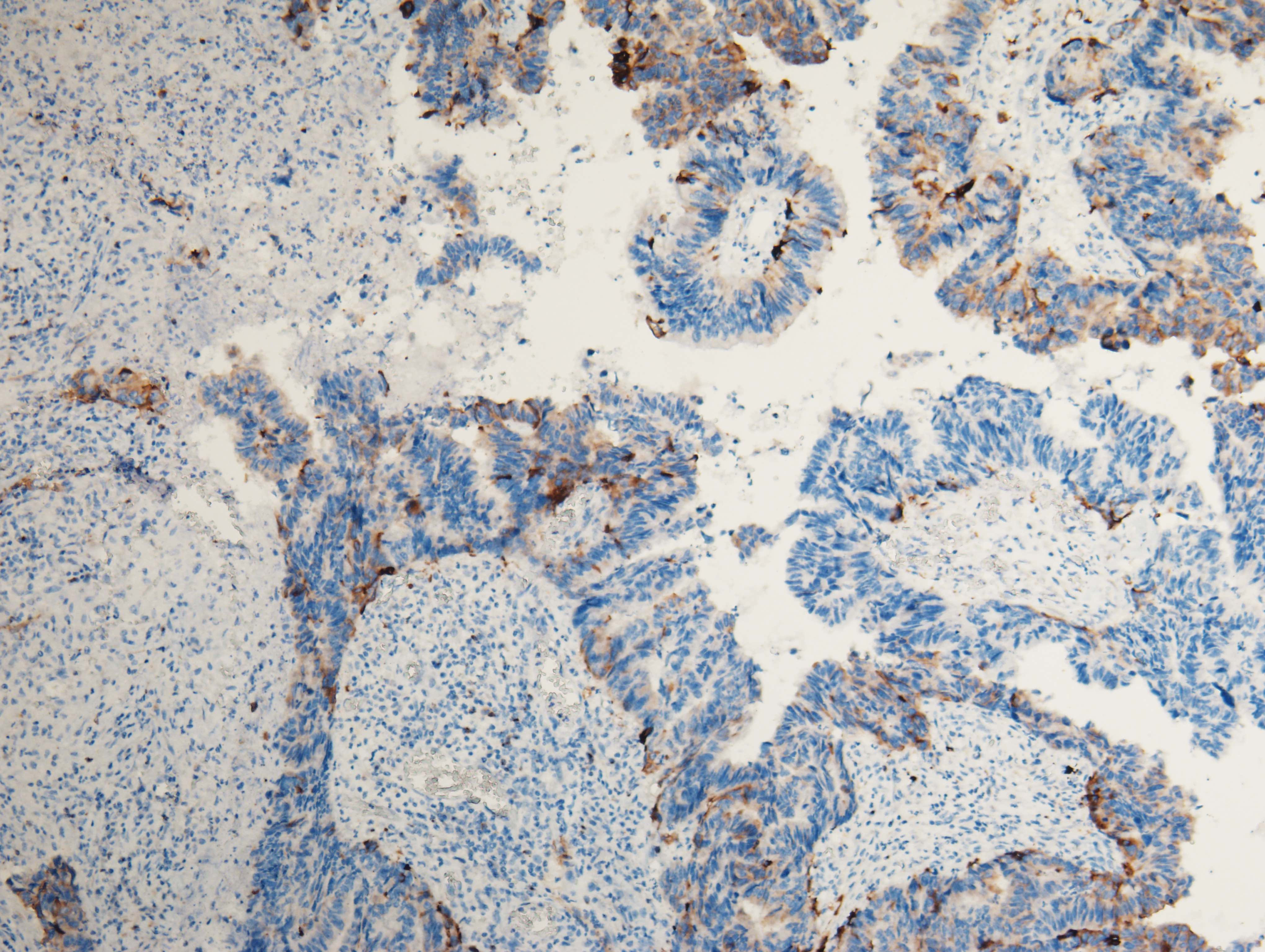

Supplement: Supplementary file 3 [file DataSheet_3.zip › OriginalImages/2-L-100x-2-1.jpeg]

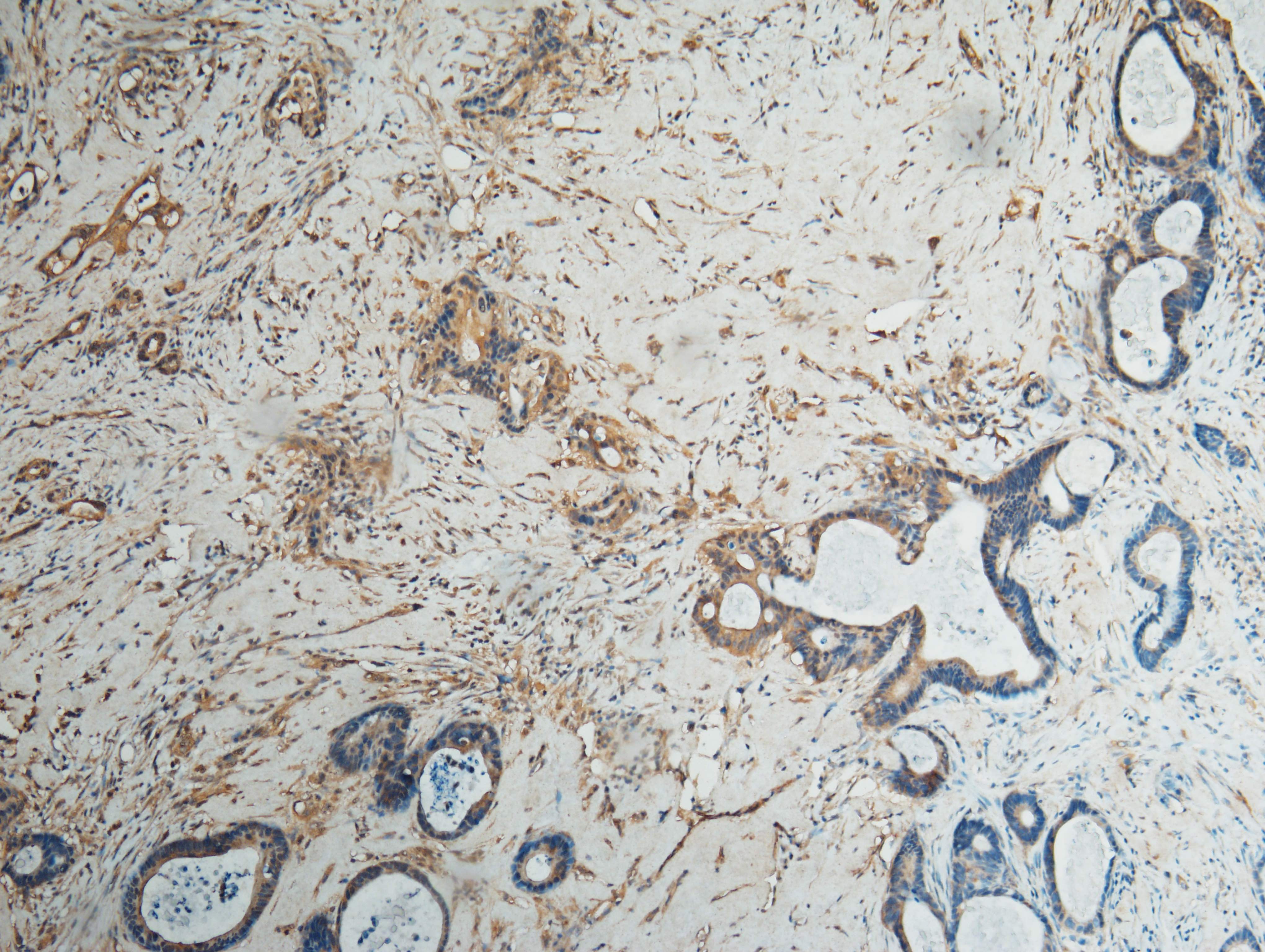

Supplement: Supplementary file 3 [file DataSheet_3.zip › OriginalImages/1-L-100x-2.jpeg]

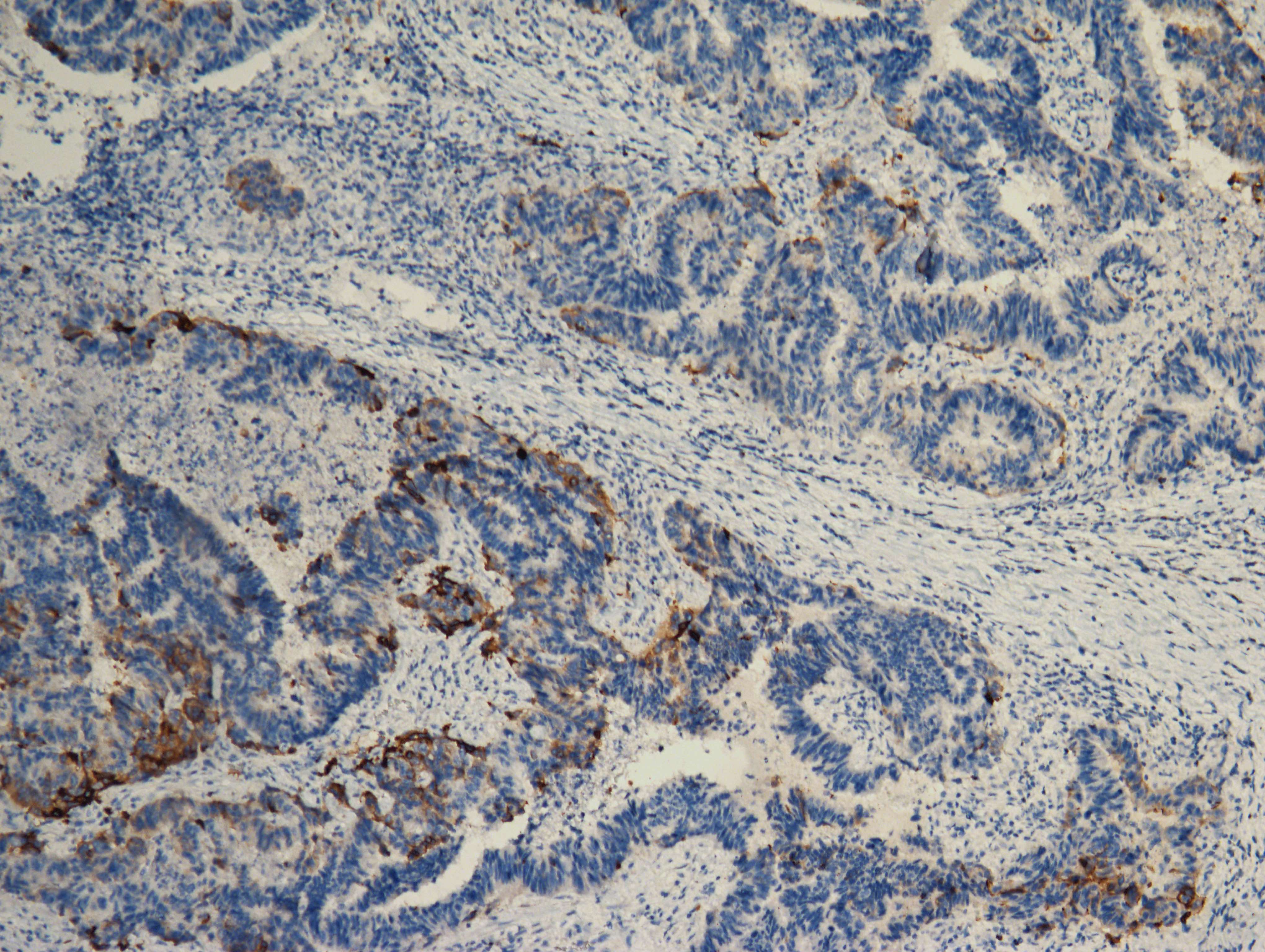

Supplement: Supplementary file 3 [file DataSheet_3.zip › OriginalImages/3-C-100x-1.jpg]

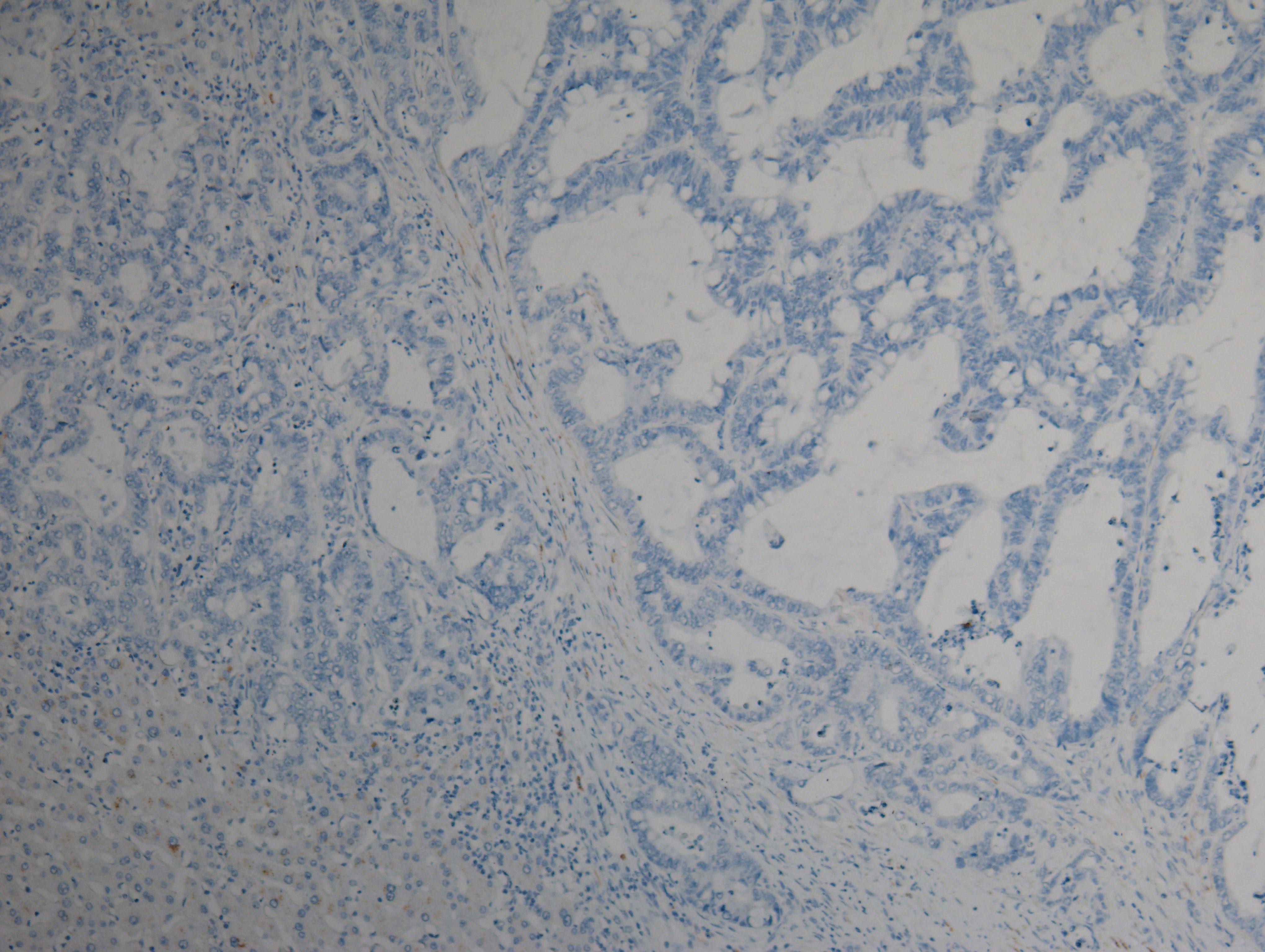

Supplement: Supplementary file 3 [file DataSheet_3.zip › OriginalImages/-L-100x-1.jpeg]

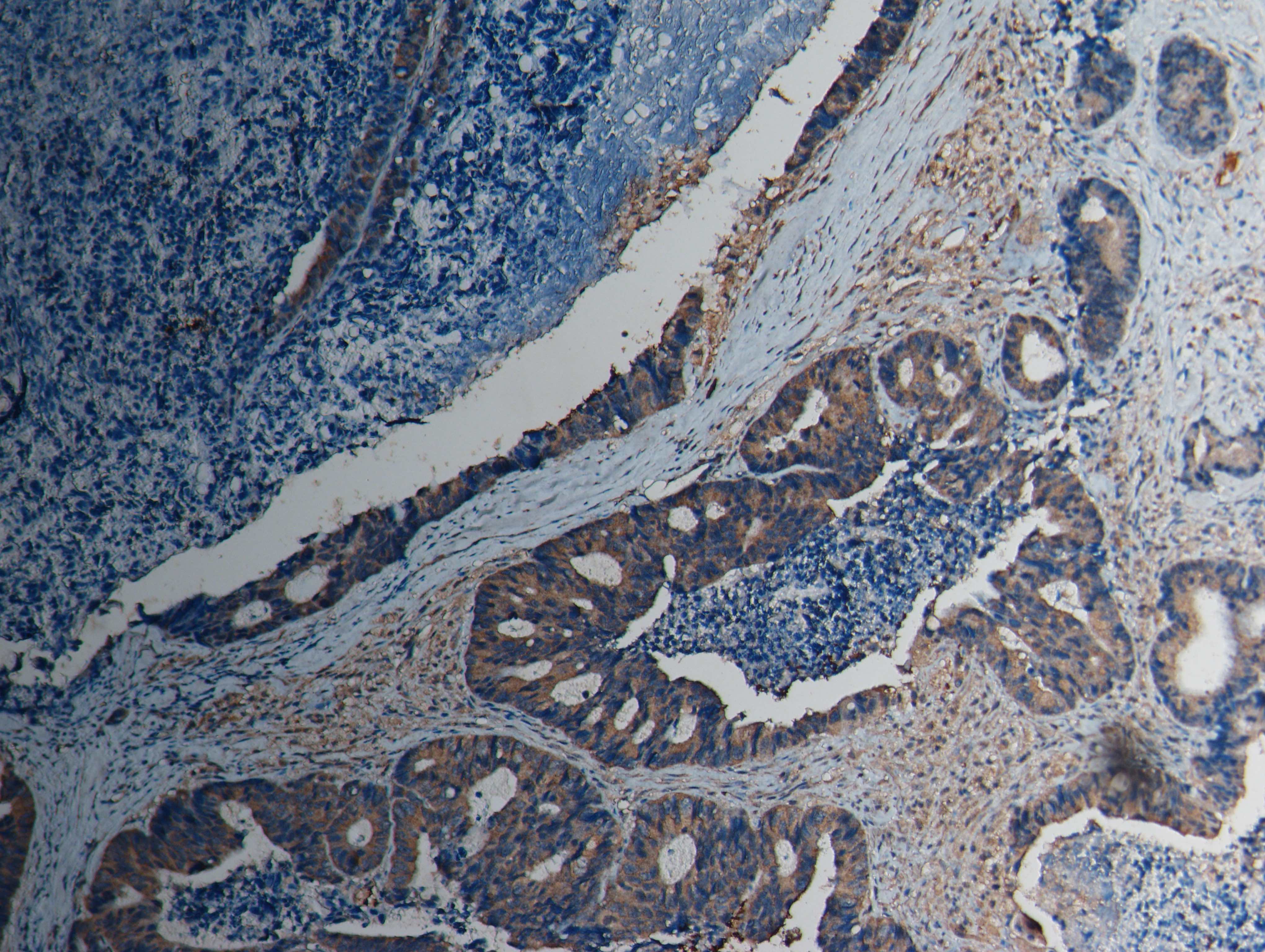

Supplement: Supplementary file 3 [file DataSheet_3.zip › OriginalImages/3-L-100x-1.jpeg]

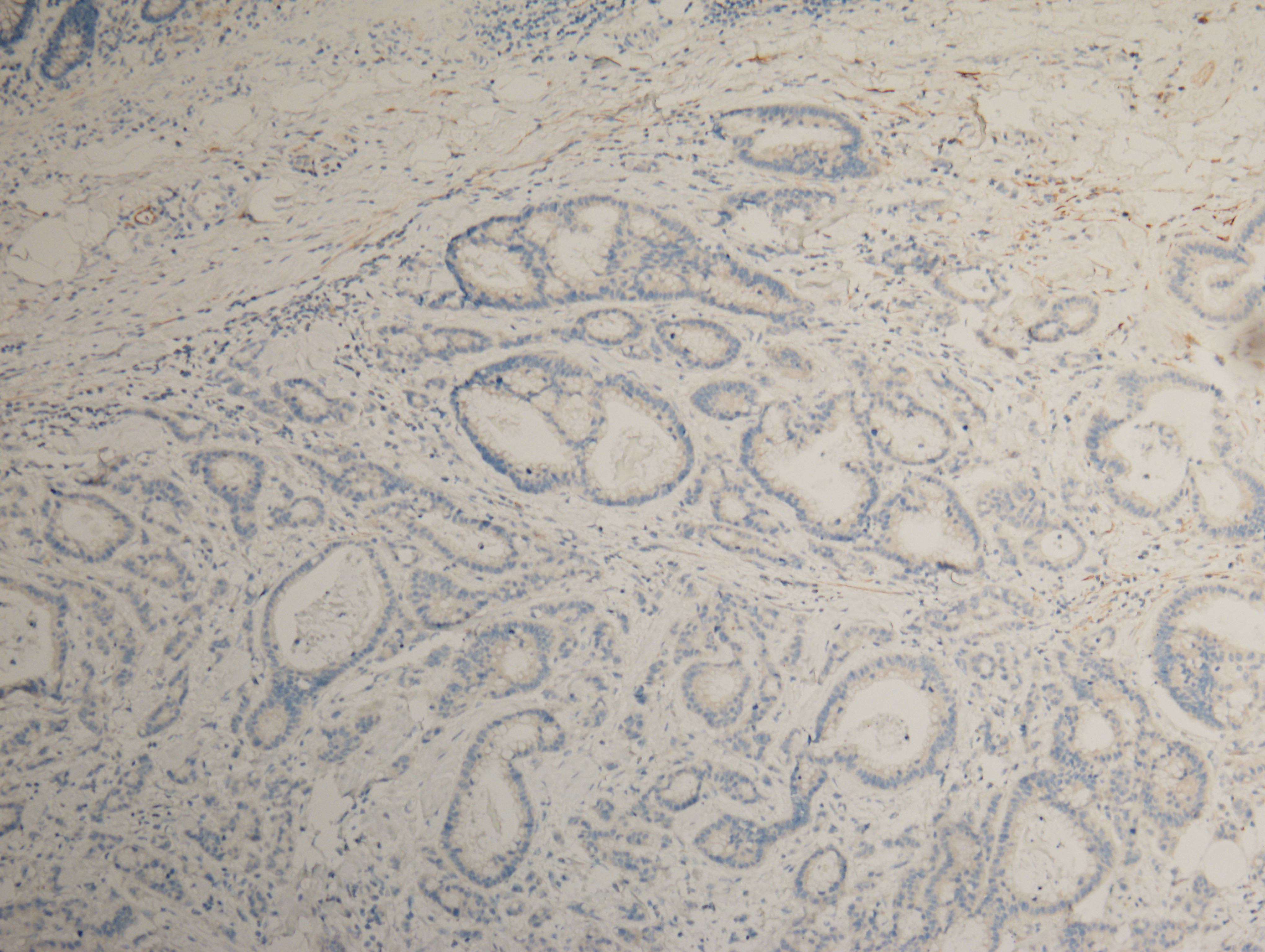

Supplement: Supplementary file 3 [file DataSheet_3.zip › OriginalImages/-C-100x-2.jpeg]

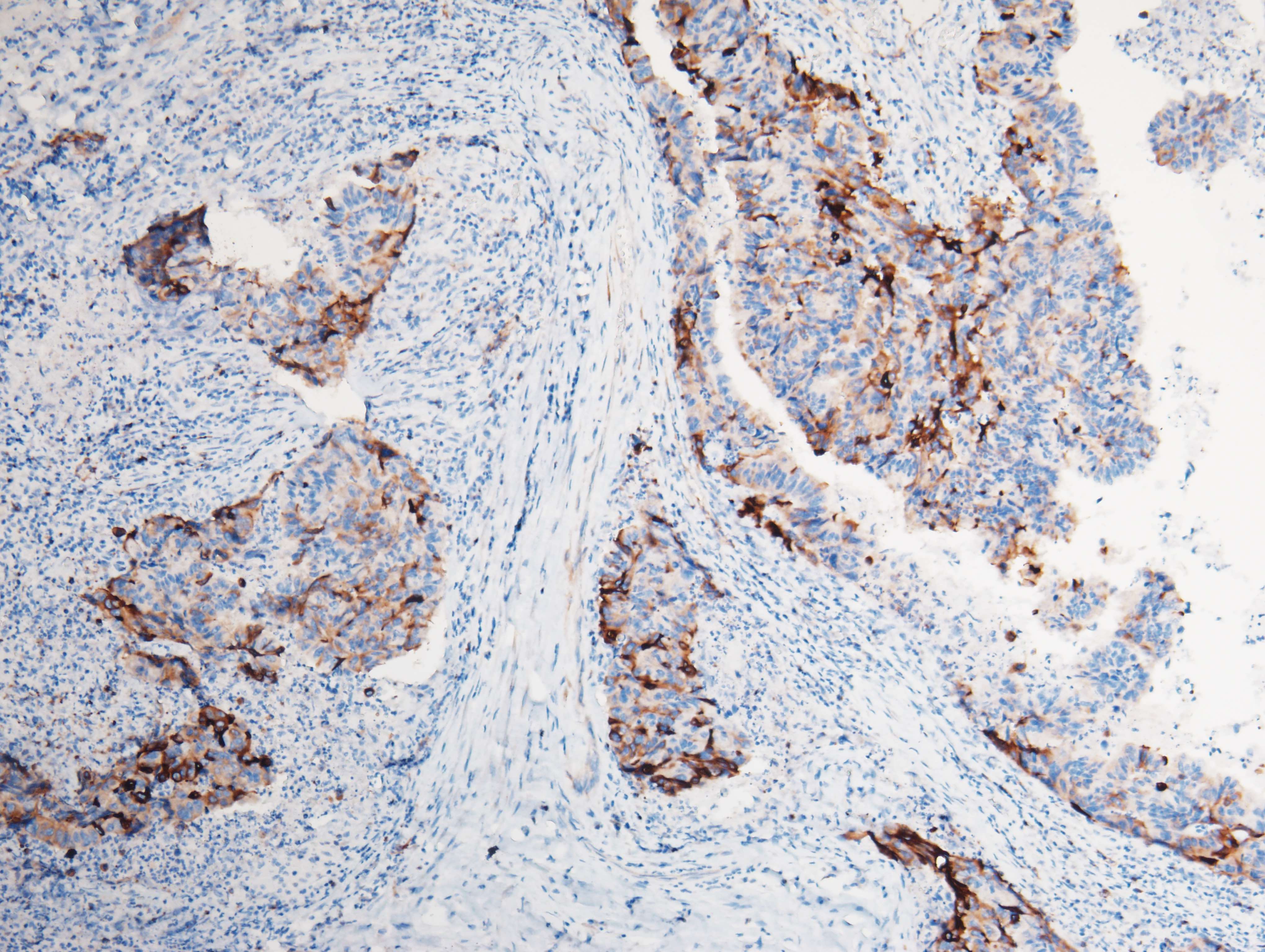

Supplement: Supplementary file 3 [file DataSheet_3.zip › OriginalImages/3-F-100x-2.jpeg]
